# Associations of Epstein-Barr Virus-Positive Gastric Adenocarcinoma with Circulating Mediators of Inflammation and Immune Response

**DOI:** 10.3390/cancers10090284

**Published:** 2018-08-23

**Authors:** M. Constanza Camargo, Armands Sivins, Sergejs Isajevs, Valdis Folkmanis, Dace Rudzīte, Margaret L. Gulley, G. Johan Offerhaus, Marcis Leja, Charles S. Rabkin

**Affiliations:** 1 Division of Cancer Epidemiology and Genetics, National Cancer Institute, National Institutes of Health, 9609 Medical Center Dr., BG 9609/6E338, Bethesda, MD 20892, USA; rabkinc@exchange.nih.gov; 2 Institute of Clinical and Preventive Medicine and Faculty of Medicine, University of Latvia, LV1586 Riga, Latvia; armands1@hotmail.com (A.S.); sergisajevs@inbox.lv (S.I.); valdis.folkmanis@lu.lv (V.F.); dacerudzite2008@inbox.lv (D.R.); cei@latnet.lv (M.L.); 3 Department of Pathology, University of North Carolina, Chapel Hill, NC 27599, USA; margaret_gulley@med.unc.edu; 4 Department of Pathology, University Medical Center Utrecht, 3508 GA Utrecht, The Netherlands; g.j.a.offerhaus@umcutrecht.nl

**Keywords:** chemokines, EBV, gastric cancer, inflammation, PD-L1

## Abstract

Epstein-Barr virus (EBV)-positive gastric adenocarcinoma exhibits locally intense inflammation but systemic manifestations are uncertain. Our study examined whether circulating mediators of inflammation and immune response differ by tumor EBV status. From a Latvian series of 302 gastric cancer cases, we measured plasma levels of 92 immune-related proteins in the 28 patients with EBV-positive tumors and 34 patients with EBV-negative tumors. Eight markers were statistically significantly higher with tumor EBV positivity: chemokine C-C motif ligand (CCL) 20 (Odds Ratio (OR) = 3.6; *p*-trend = 0.001), chemokine C-X-C motif ligand 9 (OR = 3.6; *p*-trend = 0.003), programmed death-ligand 1 (PD-L1; OR = 3.4; *p*-trend = 0.004), interleukin (IL)-10 (OR = 2.4; *p*-trend = 0.019), CCL19 (OR = 2.3; *p*-trend = 0.019), CCL11 (OR = 2.2; *p*-trend = 0.026), IL-17A (OR = 2.0; *p*-trend = 0.038) and CCL8 (OR = 1.9; *p*-trend = 0.049). Systemic responses to EBV-positive gastric cancer are characterized by alterations in chemokines and PD-L1. Profiling of these molecules may enable non-invasive diagnosis of EBV status when tumor tissue is unavailable. Our findings provide theoretical justification for clinical evaluations of immune checkpoint therapy for EBV-positive gastric cancer.

## 1. Introduction

Tumor Epstein-Barr virus (EBV) positivity defines one of four major molecular subtypes of gastric cancer in The Cancer Genome Atlas (TCGA) [1]. However, EBV status is not routinely determined in clinical practice since tailored therapies are not currently available. Histologically, EBV-positive gastric cancer is characterized by prominent inflammatory infiltrate, consisting primarily of CD8-positive or CD4-positive T cells [2,3]. We hypothesized that EBV status in cancer tissue may also be reflected in profiles of circulating mediators of inflammation and immune response.

## 2. Results

**Figure 1:**
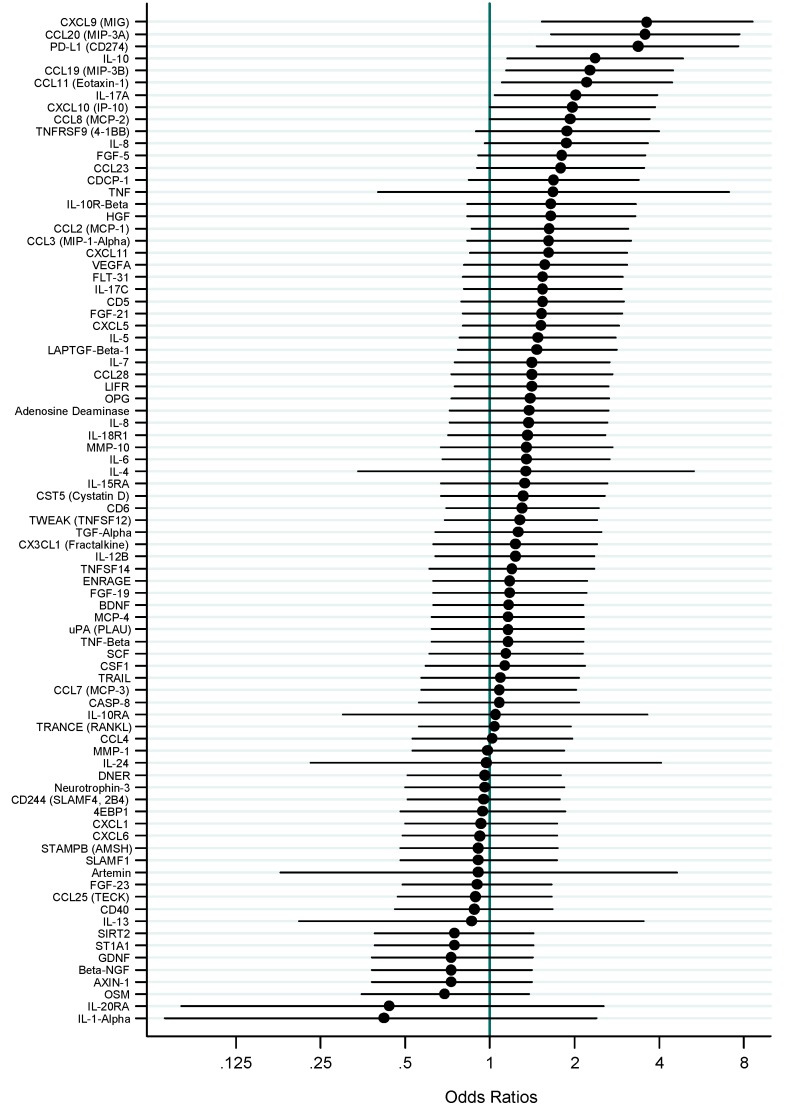
Age- and sex-adjusted odds ratios and 95% confidence intervals for associations (per quartile) between tumor EBV positivity and 83 circulating mediators of inflammation and immune response.

In line with our frequency-matched sample design, individuals with EBV-positive and -negative tumors had similar demographic and clinicopathological characteristics. Nine markers had undetectable levels in all or almost all samples. Of 83 markers evaluated (Figure 1), eight were statistically significantly higher in patients with EBV-positive tumors vs. those with EBV-negative tumors, including chemokine C-C motif ligand (CCL) 20 (per quantile Odds Ratio (OR) = 3.6; *p*-trend = 0.001), chemokine C-X-C motif ligand 9 (OR = 3.6; *p*-trend = 0.003), programmed death-ligand 1 (PD-L1; OR = 3.4; *p*-trend = 0.004), interleukin (IL)-10 (OR = 2.4; *p*-trend = 0.019), CCL19 (OR = 2.3; *p*-trend = 0.019), CCL11 (OR = 2.2; *p*-trend = 0.026), IL-17A (OR = 2.0; *p*-trend = 0.038), and CCL8 (OR = 1.9; *p*-trend = 0.049). Tertile-specific ORs and area under the receiver operating characteristic curve (AUC) are presented in the Table 1. The 28 pair-wise Spearman correlations among these eight markers ranged from 0.14 to 0.71, of which 24 were statistically significant. A model combining the top three markers, CCL20, CXCL19 and PD-L1, discriminated tumor EBV status with an AUC of 0.82.

**Figure 2:**
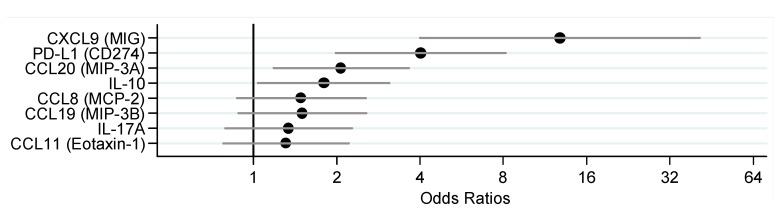
Age- and sex-adjusted odds ratios and 95% confidence intervals for associations between tumor EBV positivity and tumor mRNA expression of eight mediators of inflammation and immune response in gastric cancer from TCGA.

We next examined mRNA levels of these eight markers in tumor tissue of 24 EBV-positive and 238 EBV-negative gastric cancers in data from TCGA. Tumor EBV positivity was similarly associated with higher tissue expression of all eight markers, with statistically significant age- and sex-adjusted ORs for CCL20, CXCL19, PD-L1 and IL-10 (Figure 2).

## 3. Discussion

All but one of our statistically significant inflammation-related molecules have been implicated in host response to EBV infection and/or pathophysiology of EBV-related nasopharyngeal carcinoma (NPC) and lymphoma. CCL20 is a T-cell chemoattractant that regulates leukocyte trafficking through lymphoid tissues and sites of inflammation. This CC-chemokine is known to be up-regulated in tumor tissue of NPC and some EBV-associated lymphomas, and is induced by EBV nuclear antigen 1 in vitro [4]. Serum levels were found to be significantly higher in patients with untreated NPC, recurrent disease or distant metastases as compared to cancer-free controls, patients in remission, and long-term disease-free patients, respectively [5]. Additionally, functional assays showed that CCL20 contributed to migration and invasion of NPC cells in vitro and was effectively inhibited by specific knockdown [5]. 

CXCL9 is an interferon gamma (IFN-γ)-inducible chemokine that upon binding to its receptor CXCR3 elicits chemotactic activity on T cells. CXCL9 expression is significantly elevated in NPC tumor tissue. Moreover, circulating protein levels are associated with tumor burden and aggressiveness as well as EBV DNA load [6]. 

The programmed death-1 (PD-1) pathway is an important regulator of antimicrobial and self-reactive T cell responses and a new target in cancer immunotherapy. Elevated PD-L1 expression is a feature of NPC and other EBV-associated malignancies [7]. EBV latent membrane protein (LMP) 1 cooperates with IFN-γ pathways to regulate PD-L1 [8]. In agreement, expression of PD-L1 is suppressed by knocking down LMP1 in EBV-positive cell lines. Furthermore, higher levels of PD-L1 are associated with worse disease-free survival in NPC patients. 

IL-10 is a cytokine with strong anti-inflammatory properties that can be induced by several EBV gene products, including immediate-early protein Zta, LMP1 and EBV-encoded RNA (EBER) [9]. In B cells, IL-10 expression is enhanced by viral LMP2A which induces phosphorylation of STAT3 through activation of PI3K and BTK [10]. Circulating IL-10 levels are frequently elevated in patients with NPC as compared to healthy controls [11]. 

CCL19 is a chemokine that may play a role in normal lymphocyte recirculation and homing as well as in inflammatory and immunological responses. One of its receptors, CCR7, is induced by EBV and is thought to mediate viral effects on B lymphocytes. 

CCL11 is a chemokine that selectively recruits eosinophils. CCL11 expression is significantly higher in Hodgkin lymphoma tissue [12]. 

IL-17A is a pro-inflammatory cytokine that induces production of other cytokines, chemokines, and prostaglandins. In vitro, IL-17 production is enhanced by EBV [13]. 

Finally, CCL8 is a chemokine secreted from antigen presenting cells, for which an association with EBV remains uncertain.

## 4. Patients and Methods

We evaluated tumor EBV status for 302 gastric cancer patients consecutively treated between 2009 and 2016 at Riga East University Hospital, Latvia. Formalin-fixed paraffin-embedded tumors (in microarrays) were assessed by in situ hybridization for EBER, the gold standard assay for detecting latent infection, using a standard method as previously described [14]. A tumor was considered EBV-negative if EBER staining was undetected or only expressed in benign-appearing lymphoid cells, and EBV-positive if EBER was localized to the nucleus of malignant epithelial cells. The 28 patients with EBV-positive adenocarcinoma were frequency-matched to 34 with EBV-negative tumors by age at diagnosis (overall mean, 63 years), sex (89% males), anatomical subsite (5% cardia, 95% non-cardia) and Lauren histological type (24% diffuse, 61% intestinal, 15% mixed/unspecified). The original study was approved by the ethical committees of the Riga East University Hospital Support Foundation and of the Riga East University Hospital. All participants signed an informed consent form. The current analysis was exempted from National Institutes of Health (NIH) Institutional Review Board evaluation by the NIH Office of Human Subjects Research Protections (ID#: 17-NCI-00104, 21 April 2017). 

Pre-treatment EDTA plasma was measured for 92 protein markers on the Proseek Multiplex Inflammation I panel (Olink Proteomics, Sweden) using the Fluidigm BioMark HD real-time polymerase chain reaction platform, as described previously [15]. Values were expressed as Normalized Protein eXpression (NPX) units, which represent relative quantification of protein levels. NPX values below the lower limit of detection (LLOD) were replaced by LLOD/2. Nine markers had undetectable levels in all or almost all samples (IL-2, IL-2R-beta, IL-20, IL-22R-alpha 1, IL-33, INF-gamma, Leukemia Inhibitory Factor, Thymic Stromal Lymphopoietin, and Neurturin) and were excluded from analysis. Coefficients of variation for the remaining markers were <30%. Laboratory staff was blinded to tumor EBV status. 

The 83 evaluable proteins were analyzed as categorical variables with two or three levels based on proportion of individuals with measurements less than LLOD, as follows. Markers with <25% of individuals below the LLOD (*n* = 73) were categorized into tertiles (based on the distribution among all individuals); markers with 25% to 50% of individuals below the LLOD (*n* = 2) were categorized as less than LLOD and below and above the median; and markers with 75% to 90% of individuals below the LLOD (*n* = 8) were categorized as undetectable and detectable. For variables with three groups, tests for linear trend were performed by assigning values of 0, 1, and 2 and modeling as ordinal variables. Unconditional logistic regression was used to calculate ORs and 95% confidence intervals for the associations of the ordinal variables and individual marker tertiles with tumor EBV positivity. Models were adjusted for age and sex. AUCs were generated to assess the diagnostic accuracy of each marker. Correlations among markers were evaluated by Spearman rank correlation. *p* values <0.05 were considered statistically significant. We did not adjust for multiple comparisons because of the exploratory nature of the study. All statistical analyses were performed using STATA version 14 (Stata Corporation, College Station, TX, USA). 

## 5. Conclusions

Our study represents the first comprehensive investigation of circulating inflammation-related proteins and EBV-positive gastric cancer. These data indicate that the local alterations of specific chemokines and PD-L1 characterizing EBV-positive gastric tumors are reflected in the systemic circulation. Profiling of these molecules may enable non-invasive diagnosis of EBV-positive gastric cancer and facilitate etiologic and translational studies in large-scale prospective cohorts that lack tumor specimens. These blood-based tests may prove more generally useful for monitoring of tumor burden. Our findings also provide theoretical support for evaluating PD-1 blockade and other immunomodulatory therapies for patients with EBV-positive gastric cancer.

## Figures and Tables

**Table 1:** Circulating inflammatory proteins statistically significantly associated with tumor EBV positivity.

Protein (Other Names)	Marker Level Tertile	Age- and Sex-Adjusted OR (95% CI)
CCL20 (MIP-3A)	1	1.0
	2	2.33 (0.57–9.49)
	3	12.30 (2.68–56.36)
	*p*-trend	0.001
	AUC	0.76
CXCL9 (MIG, EBV-induced molecule 1 ligand chemokine)	1	1.0
	2	2.17 (0.44–10.81)
	3	11.49 (2.08–63.55)
	*p*-trend	0.003
	AUC	0.74
PD-L1 (CD274)	1	1.0
	2	5.80 (0.96–35.18)
	3	13.73 (2.21–85.07)
	*p*-trend	0.004
	AUC	0.73
IL-10	1	1.0
	2	2.91 (0.73–11.61)
	3	5.72 (1.33–24.65)
	*p*-trend	0.019
	AUC	0.67
CCL19 (MIP-3B)	1	1.0
	2	1.12 (0.30–4.20)
	3	5.03 (1.30–19.49)
	*p*-trend	0.019
	AUC	0.67
CCL11 (Eotaxin-1)	1	1.0
	2	1.57 (0.41–6.09)
	3	4.74 (1.18–19.02)
	*p*-trend	0.026
	AUC	0.70
IL-17A	1	1.0
	2	4.29 (1.11–16.64)
	3	4.28 (1.09–16.80)
	*p*-trend	0.038
	AUC	0.67
CCL8 (MCP-2)	1	1.0
	2	2.10 (0.56–7.88)
	3	3.72 (1.01–13.75)
	*p*-trend	0.049
	AUC	0.67

Abbreviations: OR, odds ratio; CI, confidence interval; AUC, area under the receiver operating characteristic curve.
